# Association of GSDMD with microvascular-ischemia reperfusion injury after ST-elevation myocardial infarction

**DOI:** 10.3389/fcvm.2023.1138352

**Published:** 2023-06-06

**Authors:** Wenjing Sun, Chunqiu Wang, Shihua Cui, Yan Wang, Shenghui Zhao, Min Lu, Fan Yang, Shujuan Dong, Yingjie Chu

**Affiliations:** ^1^ Department of Cardiology, Henan Provincial People's Hospital, People's Hospital of Zhengzhou University, Zhengzhou, China; ^2^ Microbiome Laboratory, Henan Provincial People's Hospital, People's Hospital of Zhengzhou University, Zhengzhou, China; ^3^ Department of Radiology, Henan Provincial People's Hospital, People's Hospital of Zhengzhou University, Zhengzhou, China; ^4^ Department of Cardiology, Dalian Medical University, Dalian, China; ^5^ Department of Cardiology, Second Affiliated Hospital of Kunming Medical University, Kunming, China

**Keywords:** ST-segment elevation myocardial infarction, magnetic resonance imaging, microvascular dysfunction, GSDMD, IMH, MVO

## Abstract

**Objectives:**

Little is known about the clinical prognosis of gasdermin D (GSDMD) in patients with ST-elevation myocardial infarction (STEMI). The purpose of this study was to investigate the association of GSDMD with microvascular injury, infarction size (IS), left ventricular ejection fraction (LVEF), and major adverse cardiac events (MACEs), in STEMI patients with primary percutaneous coronary intervention (pPCI).

**Methods:**

We retrospectively analyzed 120 prospectively enrolled STEMI patients (median age 53 years, 80% men) treated with pPCI between 2020 and 2021 who underwent serum GSDMD assessment and cardiac magnetic resonance (CMR) within 48 h post-reperfusion; CMR was also performed at one year follow-up.

**Results:**

Microvascular obstruction was observed in 37 patients (31%). GSDMD concentrations ≧ median (13 ng/L) in patients were associated with a higher risk of microvascular obstruction and IMH (46% vs. 19%, *P* = 0.003; 31% vs. 13%, *P* = 0.02, respectively), as well as with a lower LVEF both in the acute phase after infarction (35% vs. 54%, *P* < 0.001) and in the chronic phase (42% vs. 56%, *P* < 0.001), larger IS in the acute (32% vs. 15%, *P* < 0.001) and in the chronic phases (26% vs. 11%, *P* < 0.001), and larger left ventricular volumes (119 ± 20 vs. 98 ± 14, *P* = 0.003) by CMR. Univariable and multivariable Cox regression analysis results showed that patients with GSDMD concentrations ≧ median (13 ng/L) had a higher incidence of MACE (*P* < 0.05).

**Conclusions:**

High GSDMD concentrations in STEMI patients are associated with microvascular injury (including MVO and IMH), which is a powerful MACE predictor. Nevertheless, the therapeutic implications of this relation need further research.

## 1. Introduction

STEMI is the most acute manifestation of acute coronary syndrome and is associated with considerable morbidity and mortality (1). Although pPCI is the most effective approach to rescue viable myocardium, reduce infarction size, and improve cardiac function, the existence of microvascular post-reperfusion dysfunction adversely affects patient prognosis. Microvascular dysfunction [microvascular obstruction (MVO) and intramyocardial hemorrhage (IMH)] due to myocardial ischemia-reperfusion (MI/R) injury is associated with a poorer cardiac function and prognosis (2–4). Little is known about the pathophysiological processes of MVO and IMH, but inflammation has been considered to be a pivotal factor of this process, through the involvement of monocytes, neutrophils and cytokines (5, 6). In addition, the excessive inflammation after myocardial ischemia reperfusion injury could result in cell death.

Gasdermin D (GSDMD) was recently identified as a mediator of pyroptosis-inflammatory cell death triggered by cytosolic sensing of invasive infection and danger signals (7, 8). Upon activation, GSDMD forms cell membrane pores, inducing the release of pro-inflammatory cytokines and damaging the integrity of the cell membrane (9). Accumulating evidence has shown that GSDMD-mediated pyroptosis plays a pivotal role in a large number of diseases (10, 11). Our previous animal experiments indicated that GSDMD-mediated pyroptosis is involved in post-reperfusion microvascular injury (12, 13). However, whether GSDMD-medicated pyroptosis may exert its detrimental effects in STEMI patients by facilitating microvascular dysfunction remains unknown.

Cardiac magnetic resonance (CMR) imaging has been increasingly used for assessment of myocardial function, myocardial edema, infarction size (IS), and microvascular damage with high accuracy and within a single examination (14). The recent availability of multiparametric mapping CMR has provided novel insights into the pathophysiology underlying post-STEMI MVO and IMH (2, 15). Earlier research indicated that the presence of MVO and IMH was associated with higher concentrations of inflammatory factors and myocardial necrosis (15). Yet, new parametric mapping techniques enable the accurate quantification of myocardial necrosis and microvascular damage based on changes in T1, T2, and T2*(star) relaxation times and extracellular volume (ECV) (14). In addition, T2* mapping is currently considered the best method for IMH extent quantification (16, 17). Therefore, the relationship between GSDMD and T2* mapping or other CMR characteristics is a problem deserving research attention.

In the present work, CMR was used to confirm the presence of MVO and IMH, and to study myocardial function, IS, left ventricular volume. The objective of this study was to investigate the association between GSDMD in serum post-reperfusion and microvascular injury confirmed by CMR, and investigate the prognostic value of GSDMD as well as CMR parameters in post-reperfusion STEMI patients.

## 2. Material and methods

### 2.1. Study population

The present study is a retrospective, observational analysis of 120 consecutive STEMI patients enrolled between June 2020 and May 2021 in Henan Provincial People's Hospital. The follow-up duration was one year. All patients had been treated with pPCI within 12 h after symptoms onset and had undergone CMR in the early post-infarction phase (within two days from symptoms onset). Exclusion criteria were chronic MI, acute myocarditis, Killip-IV heart failure, an estimated glomerular filtration rate <30 ml/min/1.73 m^2^, and contraindications to CMR (presence of ferromagnetic implants, aneurysm clips, significant claustrophobia, severe contrast agent allergy to gadolinium, and hemodynamic instability). Blood samples of GSDMD, CRP, IL-6, and IL-1β were obtained via peripheral venipuncture 24 ± 12 h post-PCI. All patients gave informed written consent to study protocols approved by the ethics committee of our hospital.

### 2.2. Follow-up and outcome

All patients were enrolled between June 2020 and May 2021 and were followed up until the time to an event or, in the case of no event, April 2022.The primary clinical endpoint [major adverse cardiac events (MACE)] was defined as a composite of all cause death, non-fatal reinfarction, the occurrence of new heart failure and stroke (18, 19). Additionally, we compared risk stratification based on the median GSDMD levels in patients with STEMI (i.e., low-to-intermediate risk [<13 ng/L] and high risk [≧13 ng/L]).

### 2.3. Enzyme-linked immunosorbent assay (ELISA)

GSDMD (ab272463) ELISA kits were purchased from Abcam (Cambridge, UK). The concentration of cytokines was measured by the ELISA kits according to the manufacturers' instructions. The results were normalized by volume for serum samples. All samples were tested at least in triplicate.

### 2.4. Statistical analysis

All statistical analyses were carried out using SPSS (Statistical Package for the Social Sciences version 26.0, IBM, Armonk, NY, USA). Kolmogorov-Smirnov test was used to test whether the value comes from a normal distribution. The continuous data were expressed as mean ± SD and compared between groups by *t*-test and the Wilcoxon-Mann-Whitney *U*-test. Proportions were compared by chi-square test or by Fisher's exact test, as appropriate. The association of GSDMD with LVEF, IS, and microvascular injury (MVO and IMH) was evaluated in linear as well as binary multivariable regression analyses as indicated. For binary logistic regression analysis, LVEF and IS were dichotomized by clinical established cut-off values (LVEF < 50%; IS > 24% of LV; presence of MVO or IMH) MACE-free survival was estimated and depicted by the Kaplan-Meier method. GSDMD as continuous variable was dichotomized for low concentration and high concentration before log-rank test by using optimal cutoff values determined by ROC curve. To guarantee prognostic value of GSDMD for STEMI patients. Univariable and multivariable regression models were developed to investigate the potential independent association between MVO and MACE-free survival. To disclose independent predictors of MACE, all inflammatory biomarkers and CMR parameters were included, GSDMD, IL-1β, IL-6, TNF-α, CRP, CtnI, age, smoking, diabetes, anterior infarct localization as calculated in univariable regression analysis, GSDMD, IL-1β, CtnI were entered in a multivariable regression model. CMR model included the major CMR prognosis markers (LVEF, infarct size, MVO and IMH) according to literature (15). Receiver operating curve (ROC) analysis was performed to evaluate predictors of MACE. A two-side *P* < 0.05 was considered to indicate statistically significant differences.

## 3. Results

### 3.1. Study population and patient characteristics

The baseline clinical characteristics of the classified based on a threshold of GSDMD concentrations (<13 ng/L vs. ≧13 ng/L) are presented in Table 1. Patients with GSDMD levels above the median level were more likely to have diabetes (*P *= 0.001), had more often anterior infarction (*P *= 0.004), left anterior descending artery (LAD) as an infarction-related artery (*P *= 0.004), and TIMI flow 0 pre-pPCI (*P *< 0.001) occurred more often than in patients with GSDMD levels below the median.

**Table 1:** Baseline characteristics.

Variable	Total population	GSDMD < 13 ng/L	GSDMD ≥ 13 ng/L	*P* value
	(*n* = 120)	(*n* = 68)	(*n* = 52)	
Ages (Y)	54 (40–70)	51 (40–66)	59 (45–70)	0.51
Males *n*, (%)	96 (80)	47 (69)	39 (75)	0.03
Body mass index (Kg/m^2^)	26 (24–30)	25 (24–29)	27 (24–30)	0.76
Smokingn, (%)	72 (60)	40 (58)	33 (63)	0.61
Drinking *n*, (%)	58 (48)	30 (44)	27 (52)	0.21
Hypertension *n*, (%)	67 (56)	35 (51)	31 (60)	0.90
Systolic blood pressure (mmHg)	138 ± 24	133 ± 25	140 ± 23	0.37
Diastolic blood pressure (mmHg)	84 ± 19	81 ± 17	86 ± 20	0.80
Triglyceride, mmol/L	2.0 ± 1.2	1.8 ± 0.8	2.1 ± 1.6	0.15
Cholesterol, mmol/L	4.3 ± 1.1	4.1 ± 0.7	4.6 ± 1.4	0.06
LDL, mmol/L	3.3 ± 1.3	3.2 ± 1.2	3.4 ± 1.4	0.89
Diabetes *n*, (%)	40 (33)	20 (29)	19 (37)	0.001
Door to balloon time, min	180 (120–200)	160 (120–175)	200 (120–240)	0.83
Anterior Infarction *n*, (%)	68 (57)	29 (43)	39 (66)	0.004
Infarct related artery *n*, (%)				
LAD	54 (45)	21 (31)	33 (63)	0.004
LCX	17 (14)	8 (12)	9 (17)	0.38
RCA	49 (41)	28 (41)	21 (40)	0.93
Non-IRA critical stenosis *n*, (%)				
0	28 (23)	17 (25)	11 (21)	0.62
1	48 (40)	22 (32)	26 (50)	0.05
2	44 (37)	21 (31)	23 (44)	0.13
TIMI flow 0 pre-pPCI, *n* (%)	80 (67)	32 (47)	48 (92)	<0.001
TIMI flow 3 post-pPCI, *n* (%)	108 (90)	63 (93)	45 (87)	0.27
Medication at discharge				
ACEI or ARBs *n*, (%)	78 (65)	46 (68)	32 (62)	0.48
Beta-blockers *n*, (%)	84 (70)	48 (71)	36 (69)	0.87
Statins *n*, (%)	116 (97)	65 (96)	51 (98)	0.45

Values are given (%) or meantstandard deviation. LDL, Low-density lipoprotein; LAD, left anterior descending; LCX, left circumflex; RCA, right coronary artery.

### 3.2. GSDMD and cardiac magnetic resonance parameters

**Figure 1:**
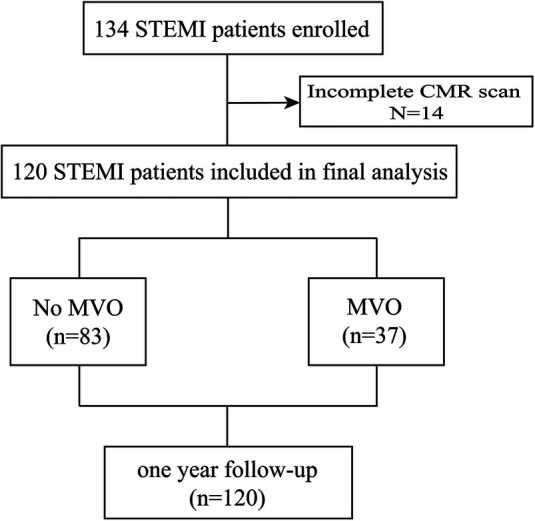
Flow diagram of the cohort study.

**Figure 2:**
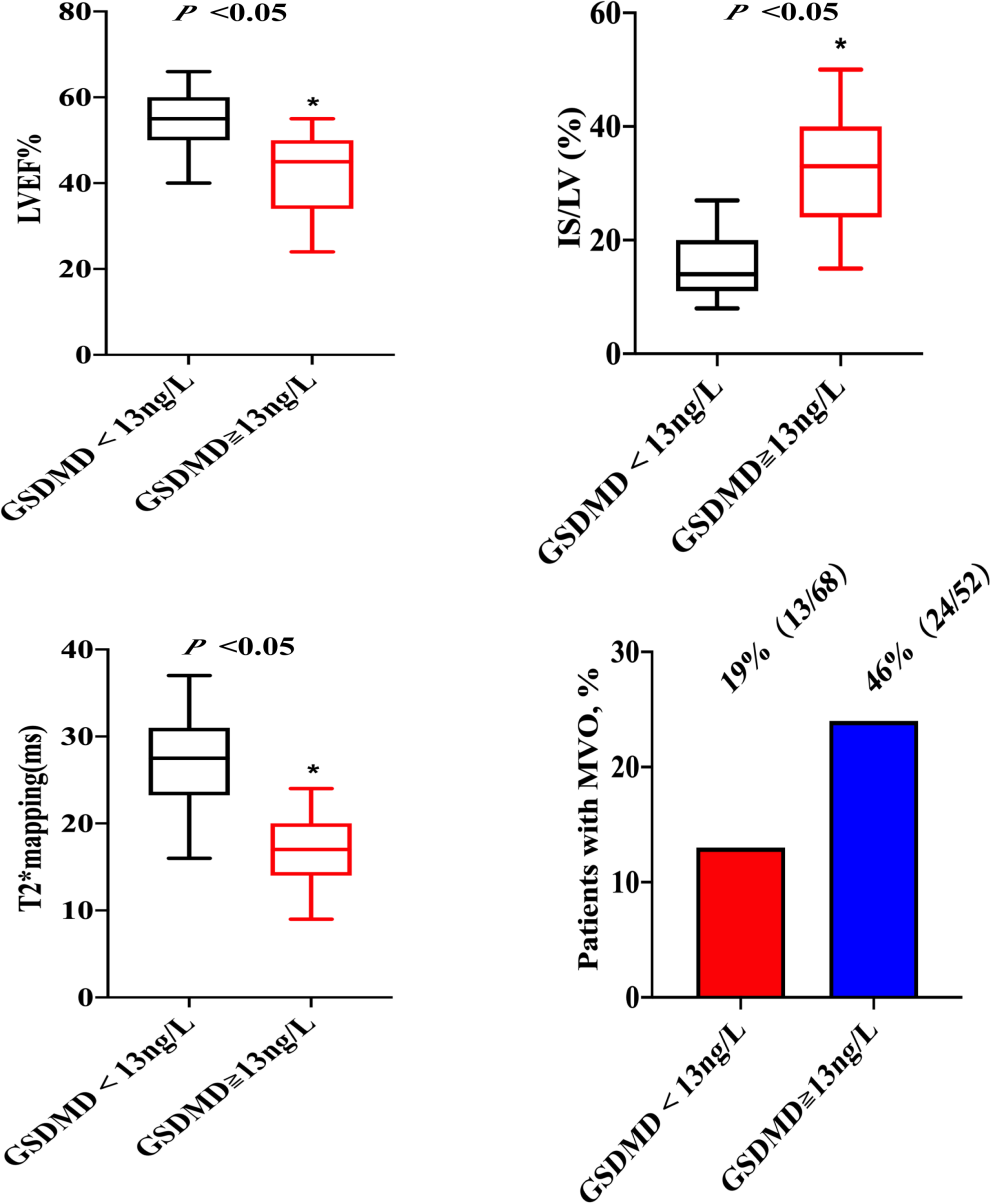
Association of GSDMD with LVEF, IS, T2*maps and MVO. LVEF, left ventricular ejection fraction; IS, infarction size; MVO, microvascular obstruction.

**Figure 3:**
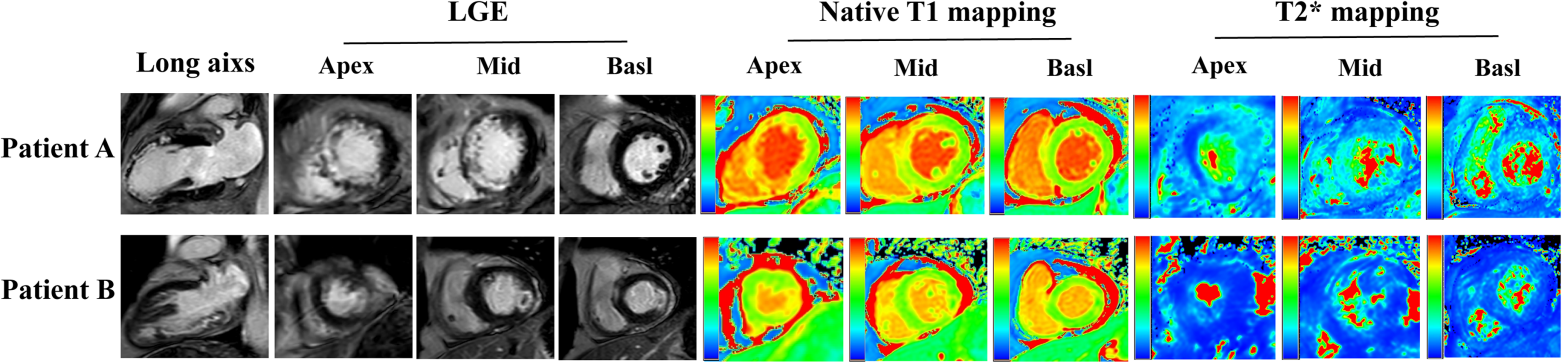
CMR images from two patients with STEMI treated by PPCI. Short axis slices in the basal, middle and apical region and long axis long axis two-chamber heart are showed. Patient A: A 57-year-old patient with anterior STEMI treated by PPCI and with GSDMD concentrations ≧ median (13 ng/L). The CMR images on day 2 showing transmural infarction in the anterior wall with MVO, and the Native T1 and T2* maps represent myocardial damage and intramyocardial hemorrhage. Patient B: A 49-year-old patient with lateral STEMI treated by pPCI and with GSDMD concentrations< median (13ng/L). The CMR images on day 2 manifesting infarction in the lateral wall with MVO, and the Native T1 and T2* maps represent myocardial damage and intramyocardial hemorrhage.

Baseline CMR characteristics and their relationship with GSDMD levels are presented in Table 2 and Table 3. Out of 134 STEMI patients enrolled, 120 (90%) had complete CMR data at the follow up examination and a median CMR time of 24 h (Figure 1). A higher risk of microvascular obstruction and IMH (46% vs. 19%, *P* = 0.003; 31% vs. 13%, *P* = 0.02, respectively) was found in patients with high GSDMD concentrations (≧13 ng/L). Furthermore, patients with high GSDMD concentrations (≧13 ng/L) showed a lower LVEF both in the acute phase after infarction (35% vs. 54%, *P* < 0.001) (Figure 2) and in the chronic phase (42% vs. 56%, *P* < 0.001). Patients presenting GSDMD concentrations ≧ median also were significantly associated with larger IS in the acute (32% vs. 15%, *P* < 0.001) and in the chronic phases (26% vs. 11%, *P* < 0.001) (Figure 2). In addition, increased GSDMD levels (≧13 ng/L) were associated with more dilated LVESV (66 ± 13 vs. 53 ± 10, *P* = 0.005) as well as LVEDV (119 ± 20 vs. 98 ± 14, *P* = 0.003) (Figure 3).

**Table 2:** Cardiac magnetic resonance parameters.

Variable	Total population	GSDMD < 13 ng/L	GSDMD ≥ 13 ng/L	*P* value
	(*n* = 120)	(*n* = 68)	(*n* = 52)	
LVEF, %	45 ± 12	54 ± 7	35 ± 9	<0.001
LVESV, ml	60 ± 14	53 ± 10	66 ± 13	0.005
LVEDV, ml	108 ± 20	98 ± 14	119 ± 20 119 ± 20	0.003
IS% of LVMM	25 ± 12		32 ± 10	<0.001
MVO, *n* (%)	37 (31)	13 (19)	24 (46)	0.003
IMH, *n* (%)	25 (21)	9 (13)	16 (31)	0.02
T1-mapping				
T1 infarct (ms)	1467 ± 118	1370 ± 50	1564 ± 79	0.01
T1 remote (ms)	1240 ± 28	1234 ± 26	1247 ± 27	0.38
T2*-mapping				
T2*infarct (ms)	16 ± 6.1	21 ± 3.5	11 ± 2.2	0.03
T2*remote (ms)	30 ± 3.9	31 ± 3.5	29 ± 4.4	0.48

Values are given (%) or mean ± standard deviation. LVEF, left ventricular ejection fraction; LVEDV, left ventricular end-diastolic volume; LVESV, left ventricular end- systolic volume; IS, infarction size; MVO, microvascular obstruction; IMH, intramyocardial hemorrhage.

**Table 3:** Cardiac magnetic resonance parameters for one-year following up.

Variable	GSDMD < 13 ng/L	GSDMD ≥ 13 ng/L	*P* value
	(*n* = 68)	(*n* = 52)	
LVEF, %	58 ± 11		<0.001
LVESV, ml	51 ± 9	65 ± 12	0.013
LVEDV, ml	94 ± 12	117 ± 14	0.006
IS% of LVMM	14 ± 8		<0.001
T1-mapping			
T1 infarct (ms)	1355 ± 48	1660 ± 73	0.01
T1 remote (ms)	1220 ± 30	1256 ± 47	0.42
T2*-mapping			
T2*infarct (ms)	24 ± 2.8	10 ± 1.4	0.02
T2*remote (ms)	36 ± 3.9	30 ± 3.2	0.39

Values are given (%) or meantstandard deviation. LVEF, left ventricular ejection fraction; LVEDV, left ventricular end-diastolic volume; LVESV, left ventricular end-systolic volume; IS, infarction size.

In multiple linear regression analysis, GSDMD was significantly related to baseline LVEF (β = −0.409, *P *< 0.001), IS (β = 0.417, *P *< 0.001), MVO (β = 0.414, *P *< 0.001), and IMH (β = 0.502, *P *< 0.001). Four models were evaluated where either baseline LVEF, IS, MVO or IMH were regarded as the dependent variable, and GSDMD, IL-1β, CtnI, diabetes, CRP and anterior infarction localization as independent variables. In all four models, plasma GSDMD levels were significantly correlated with LVEF (β = −0.357, *P *< 0.001), IS (β = 0.206, *P* = 0.017), MVO (β = 0.323, *P *< 0.001) as well as IMH (β = 0.422, *P *< 0.001, Table 4). All parameters included in multivariable analysis showed a VIF of <2.

**Table 4:** Uni-and multivariable linear regression.

	Univariable		Multivariable	
	β	*P*-value	β	*P*-value
LVEF (%)				
GSDMD, pg/ml	−0.409	<0.001	−0.357	<0.001
IL-1β, pg/ml	−0.384	<0.001	−0.296	0.003
IL-6, pg/ml	−0.226	0.039	−0.117	0.413
TNF-a, pg/ml	−0.086	0.213	–	–
CRP, mg/L	−0.297	0.003	−0.038	0.513
Ctnl, ng/ml	−0.424	<0.001	−0.377	<0.001
Age	−0.136	0.047	–	–
Smoking	−0.094	0.203	–	–
Diabetes	−0.102	0.277	–	–
Anterior infarct localization	−0.342	<0.001	−0.219	0.003
IS				
GSDMD, pg/ml	0.417	<0.001	0.206	0.017
IL-1β, pg/ml	0.388	<0.001	0.154	0.036
IL-6, pg/ml	0.303	0.002	0.113	0.154
TNF-a, pg/ml	0.085	0.109	–	–
CRP, mg/L	0.167	0.013	0.089	0.437
Ctnl, ng/ml	0.502	<0.001	0.399	<0.001
Age	0.122	0.058		–
Smoking	0.109	0.239	–	–
Diabetes	0.098	0.306	–	–
Anterior infarct localization	0.303	<0.001	0.211	0.003
MVO				
GSDMD, pg/ml	0.414	<0.001	0.323	<0.001
IL-1β, pg/ml	0.338	<0.001	0.198	0.022
IL-6, pg/ml	0.236	0.012	0.109	0.425
TNF-a, pg/ml	0.102	0.215	0.056	0.691
CRP, mg/L	0.184	0.041	0.073	0.540
Ctnl, ng/ml	0.547	<0.001	0.399	<0.001
Age	0.082	0.352	–	–
Smoking	0.096	0.215	–	–
Diabetes	0.293	0.003	0.203	0.004
Anterior infarct localization	0.159	0.177	0.062	0.317
IMH				
GSDMD, pg/ml	0.502	<0.001	0.422	<0.001
IL-1β, pg/ml	0.432	<0.001	0.293	0.014
IL-6, pg/ml	0.211	0.027	0.126	0.224
TNF-a, pg/ml	0.117	0.333	0.068	0.540
CRP, mg/L	0.341	<0.001	0.233	0.002
Ctnl, ng/ml	0.499	<0.001	0.311	<0.001
Age	0.101	0.453	–	–
Smoking	0.054	0.616		
Diabetes	0.080	0.407	–	–
Anterior infarct localization	0.113	0.129	0.055	0.403

GSDMD, Gasdermin D; IL-1β, interleukin-1β; CRP, C-reactive protein; IL-6, interleukin-6; TNF-α, tumor necrosis factor-α; LVEF, left ventricular ejection fraction; IS, infarction size; MVO, microvascular obstruction; IMH, intramyocardial hemorrhage.

In binary logistic regression analysis, GSDMD emerged as independent predictor of baseline LVEF [odds ratio (OR): 1.06, 95% CI 0.90–2.23, *P *< 0.01], IS (OR:3.29, 95% CI 1.42–5.40, *P *< 0.01), MVO (OR: 1.20, 95% CI 1.30–2.17, *P *< 0.01), and IMH (OR: 1.73, 95% CI 1.17–5.69, *P *< 0.01).

### 3.3. Association between GSDMD and native T1 and T2* mapping

On native T1 maps, the mean T1 value of the infarcted was significantly higher than that of the remote myocardium (1467 ± 118 ms vs. 1240 ± 28 ms, *P *< 0.05) (Figure 3, Table 2). Meanwhile, the mean T1 values of infarcted myocardium were higher in patients with high GSDMD concentrations (≧ 13 ng/L) both in the acute and chronic phase after infarction (1564 ± 79 ms vs. 1370 ± 50 ms, *P* = 0.01; 1660 ± 73 ms vs. 1335 ± 48 ms, *P* = 0.01) (Figure 3, Table 3). On T2* maps, the mean T2* values of infarcted myocardium were significantly lower than that of the remote area (16 ± 6.1 ms vs. 30 ± 3.9 ms, *P *< 0.05). Moreover, the mean T2* values of infarcted myocardium were lower in patients with high GSDMD concentrations (≧13 ng/L) both in the acute and chronic phase (11 ± 2.2 ms vs. 21 ± 3.5 ms, *P* = 0.03; 10 ± 1.4 ms vs. 24 ± 2.8 ms, *P* = 0.02) (Figures 2, 3, Table 3).

### 3.4. GSDMD and clinical outcome

**Figure 4:**
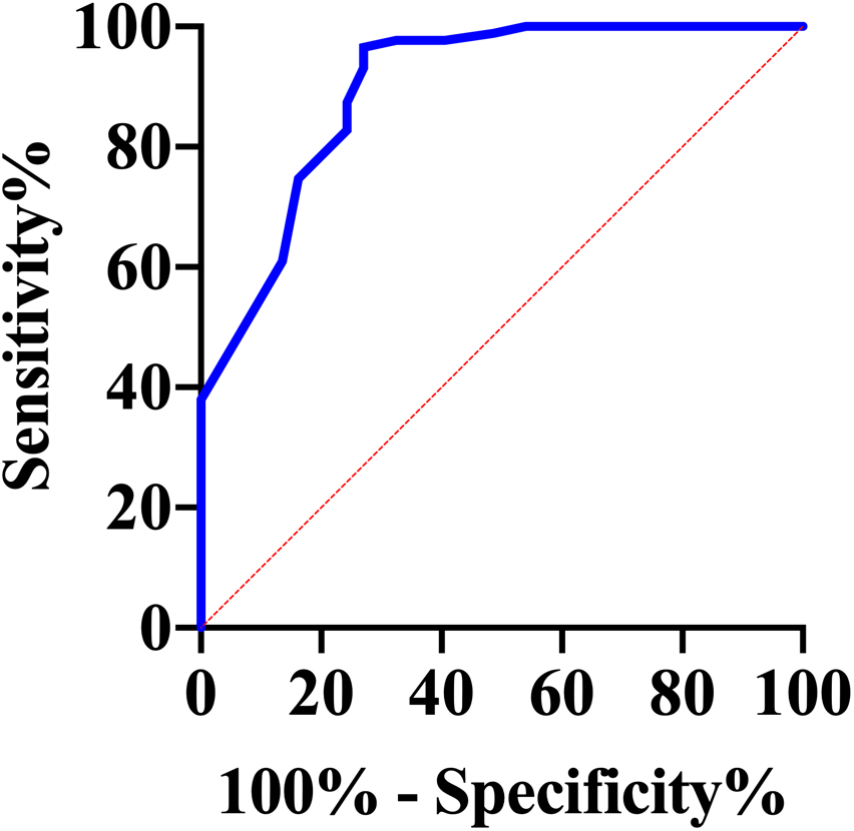
Receiver operating characteristic curve for the prediction of death in hospital in STEMI patients.

**Figure 5:**
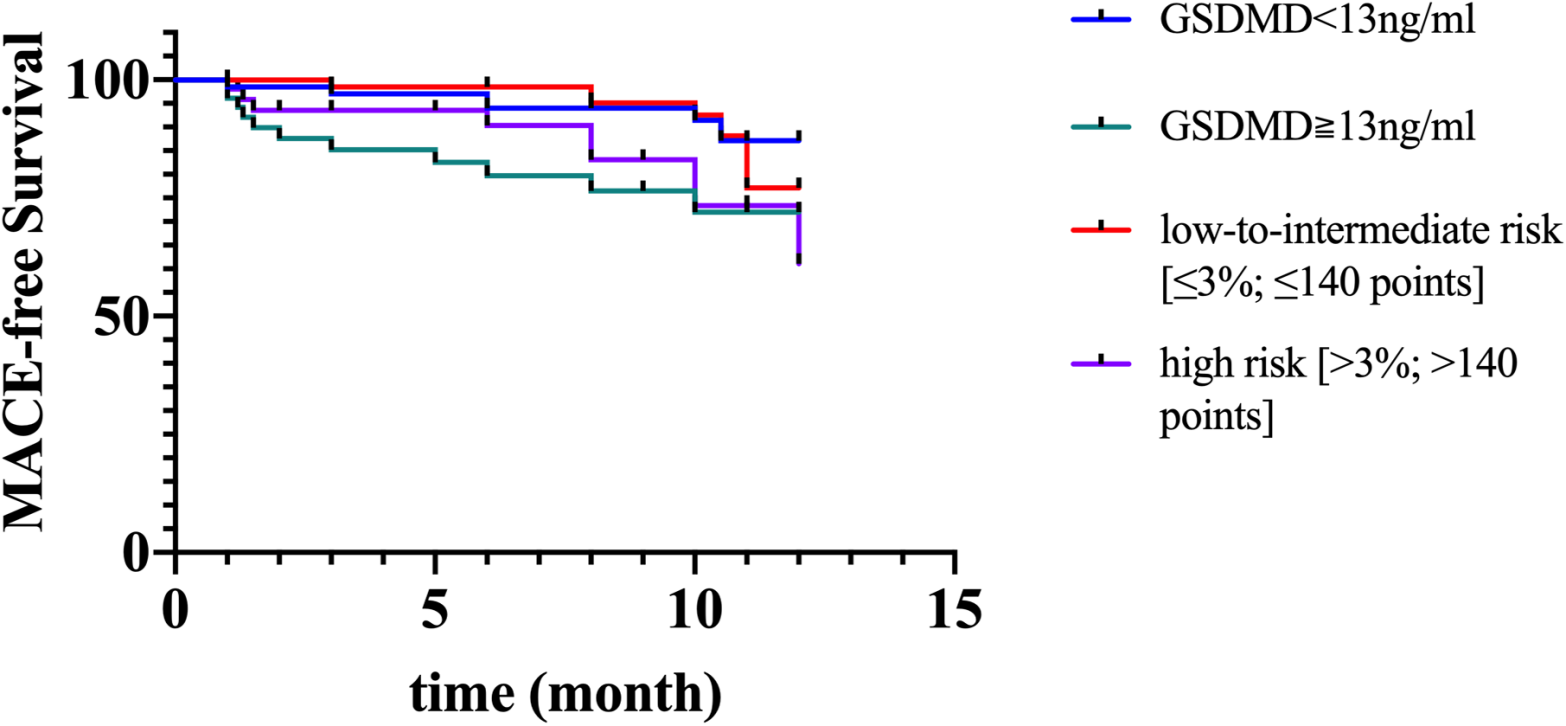
Kaplan-Meier curves suggesting the risk of MACE in relation to GSDMD concentrations compared to GRACE score. MACE, major adverse cardiac events; GSDMD, gasdermin D.

The GRACE 3.0 score has been validated for risk stratification in patients with non-ST-segment elevation acute coronary syndromes (NSTE-ACS) (20). However, the GSDMD to predict MACE in STEMI patients was unclear. MACE data after a follow-up of one-year following STEMI were available for 120 (100%) patients. Of them, 17 (14%) STEMI patients experienced MACE, including five (4%) nonfatal MI, six new congestive heart failures (5%), four unstable angina pectoris requiring revascularization (3%), and two ventricular arrhythmias (2%) occurred. In ROC analysis, circulating GSDMD significantly predicted MACE (AUC = 0.89, 95% CI 0.83–0.96, *P *= 0.02) (Figure 4). Kaplan-Meier curve analysis indicated that patients having GSDMD concentrations > median had a significantly higher MACE-free survival (hazard ratio: 3.22, [95% CI, 1.29–9.49], *P = *0.008), and there has no significance between patients with GSDMD concentrations ≧ median (13 ng/L) and high risk [>3%; >140 points] (Figure 5). After adjusting for baseline characteristics and clinical predictors, there was a 9% higher risk of MACE (adjusted hazard ratio [adj HR] 1.10, 95% CI 1.13–1.27), 12% higher risk of nonfatal MI (adj HR 1.15, 95% CI 1.07–1.31), and a 16% higher risk of congestive heart failures or ventricular arrhythmias (adj HR 1.08, 95% CI 1.01–1.24) for higher GSDMD concentrations (≧13 ng/L) patients.

**Figure 6:**
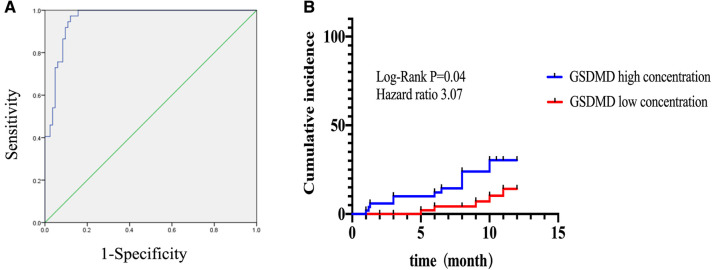
Kaplan-Meier curves according to GSDMD optimal cutoff values for predicting MACE. (**A**) Receiver operating characteristic curve for the prediction optimal cutoff values of GSDMD and for prediction microvascular dysfunction diagnosis. (**B**) High concentration of GSDMD correlated with poor outcomes in STEMI patients.

In ROC analysis, circulating GSDMD significantly predicted microvascular injury (AUC = 0.95, 95% CI 0.93–0.99, *P* = 0.0001). Furthermore, for the prediction of MACE, a cut-off value of 13.835 was set for risk assessment. Patients having high GSDMD concentration showed a significantly higher MACE-free survival, as determined by Kaplan-Meier curve analysis (Figure 6).

### 3.5. CMR and clinical outcome

During the follow-up period, the occurrence of MACE was significantly higher in patients with higher GSDMD concentrations (21% vs. 8%, *P *< 0.001). In univariable analysis, presence of MVO, presence of IMH, IS, LVEF, EDV and ESV were significantly associated with the occurrence of MACE (hazard ratio 5.93, [95% CI, 2.33–15.09], *P *< 0.001; (hazard ratio: 4.82, [95% CI, 1.78–13.00], *P = *0.002; (hazard ratio: 4.92, [95% CI, 2.02–11.98], *P *< 0.001; hazard ratio: 4.96, [95% CI, 1.83–13.48], *P = *0.002; (hazard ratio: 4.06, [95% CI, 1.52–10.28], *P = *0.003; and hazard ratio: 4.10 [95% CI, 1.61–10.42], *P *= 0.003; respectively). In the multivariable Cox regression analysis, the presence of MVO and IMH were independent predictors of a higher incidence of MACE (hazard ratio: 5.35, [95% CI, 1.35–10.27]; *P *= 0.02 and hazard ratio: 3.97, [95% CI, 1.59–8.76], *P *= 0.03; respectively) (Supplementary Table S1).

## 4. Discussion

The main findings of our study were as follows: (1) The increased levels of the GSDMD in serum were associated with failed microvascular reperfusion (including MVO and IMH), larger IS, and worse myocardial function; (2) MVO and IMH were independently associated with MACE at one-year post-STEMI. The significant association persisted after adjustment for potential biomarkers, such as IL-6, IL-1β, CRP; (3) Higher levels of circulating GSDMD were associated with a higher incidence of MVO and IMH and lower MACE-free survival. Therefore, GSDMD has a promising role for GSDMD as a prognostic biomarker for more serious outcomes in the early post-STEMI phase and could also serve as a potential target for future clinical cardio-protection trials.

While the timely restoration of the epicardial coronary artery to the ischemic myocardium by PCI is the primary approach to preserve myocardial viability, it also contributes to I/R injury not only to cardiomyocytes, but also to various myocardial components, including the microvasculature. The presence of MVO and IMH after STEMI is associated with a worse outcome (21). Accumulating evidence has revealed that inflammation and cell death are the hallmarks of post-reperfusion microvascular injury. However, the complex pathophysiology of this process has not been fully elucidated so far.

Inflammation, traditional biomarkers such as C-reactive protein (CRP), Interleukin-6 (IL-6), and interleukin-1β (IL-1β), has been associated with cardiovascular events. IL-6 is an inflammatory cytokine that mediates both immune and inflammatory responses. A body of evidence previously highlighted that IL-6 increases substantially after AMI and is involved in the pathophysiological process of microvascular injury (22). Consistent with the previous result, although a significant association between elevated IL-6 and microvascular injury has been found, this observation was not confirmed in multivariable regression analysis in our present study.

Previous studies indicated that CRP is the most extensively investigated inflammatory marker for coronary heart disease (23). Reindl et al. have been found that the concentrations of CRP were significantly higher in STEMI patients with MVO (24). Our study confirms previous experimental results, CRP were markedly associated with the occurrence of microvascular injury (MVO and IMH). Nevertheless, the relationship of CRP and microvascular obstruction was not verified by multivariable regression analysis.

High-sensitivity cardiac troponin I (hs-cTnI) assays have been recognized for improving diagnostic accuracy in the early detection of AMI and for their potential in risk stratification for patients with AMI. Previous studies demonstrate that risk assessment in STEMI patients could be improved by combination with cTnI (25). Consistent with the previous studies, despite its association with the risk of microvascular injury, this observation was not confirmed in multivariable regression analysis in our present study.

However, whether there are novel biomarkers should be explored. GSDMD mediated-pyroptosis act as a novel proinflammatory programmed cell death as well as release of proinflammatory cytokines IL-1β and IL-18 (8). Recently, roles for pyroptosis in cardiovascular disease are emerging, including myocardial infarction, ischemia-reperfusion injury, atherosclerosis and diabetic cardiomyopathy (26). However, it is not clear whether pyroptosis is associated with microvascular dysfunction following myocardial reperfusion. A recent report from our animal experiment demonstrated that I/R induced pyroptosis was involved in the pathophysiological process of microvascular dysfunction (12). Nevertheless, whether GSDMD mediated-pyroptosis contributes to microvascular dysfunction in STEMI patients is largely unknown. In the present study, we propose circulating GSDMD as a novel microvascular injury predictor post-STEMI. With the present data, relation of GSDMD with both MVO and IMH remained independent from conventional prognostic biomarkers even after adjustment for other established clinical parameters.

IL-1β acts as pro-inflammatory multifunctional cytokine and is one of the main regulators following cardiovascular events. To investigate whether IL-1β is implicated in pathophysiology of microvascular dysfunction following myocardial reperfusion. The present study revealed that IL-1β is an important contributor of microvascular injury (MVO and IMH). Since GSDMD and IL-1β are closely related, it is important to evaluate whether GSDMD provides additional prognostic value above and beyond IL-1β. In our study, we emphasize the pivotal role of GSDMD in the acute setting post infarction since the association of GSDMD with microvascular injury remained independent even after adjustment for IL-1β.

Accumulating evidence has revealed that the presence of microvascular injury (including MVO and IMH) and blood stasis adversely affects early left ventricular remodeling and are independently associated with adverse cardiovascular events (27, 28). Recently, CMR was identified as a crucial noninvasive imaging modality for assessing the cardiac morphology, function, and myocardial necrosis in STEMI patients (14). However, the association between GSDMD and CMR-derived indexes in STEMI patients is not well understood. Our data suggest that increased GSDMD levels in the serum exert negative effects on LVEF and are associated with a larger infarction and left ventricular volume assessed by CMR. However, the implementation of delayed enhancement cardiovascular magnetic resonance imaging (DE-MRI) for the assessment of acute myocardial infarction is partly hampered by methodological and technical challenges. T1 mapping has recently emerged as a promising technique for the quantification of myocardial injury early after STEMI and prediction of recovery (29, 30). In our present study, native T1 mapping could detect myocardial injury in patients with STEMI, with the results are consistent with those of a previous study (29). Interestingly, native T1 values were higher in patients with high GSDMD concentrations (≧13 ng/L), which further indicates that elevated serum GSDMD levels might be involved in the pathophysiological process of ischemia-reperfusion injury in STEMI patients who undergo pPCI.

Clinical studies have also revealed that CMR can be applied as a tool for risk stratification and guide in the management of STEMI patients at risk of MACE (31, 32). In this study, we focused on the direct impact of IS, LVEF, EDV, ESV, the presence of MVO, and IMH on the primary endpoint. Our present findings showed that MVO and IMH are the strongest predictor of the clinical outcome, which remained an independent predictor of MACE in the multivariable Cox regression analysis results after adjustment for other established prognostic risk factors including IS, LVEF, EDV and ESV.

Yet, the pathophysiological mechanisms of the relationship between MVO or IMH and worse outcome are not fully understood. Recent experimental and clinical studies have indicated the presence of a link between IMH and the persistence of inflammation (5, 33). In an animal model of myocardial reperfusion injury, massive inflammatory cells recruitment was detected in the infarction area, accompanied by MVO and IMH (34). In STEMI post-reperfusion patients, the quantification of IMH by T2* imaging found iron depositions, which was associated with an inflammatory reaction (15). Therefore, we aimed to determine whether the increased GSDMD levels in STEMI patients receiving PCI are related to MVO or IMH. Our data showed that patients with high GSDMD concentrations (≧13 ng/L) had lower T2^*^ values in the infarct region, suggesting that GSDMD may be involved in the pathophysiological processes of MVO or IMH, affecting the prognosis of post-reperfusion STEMI patients. In the present study, we confirm the prognostic significance of elevated GSDMD levels, which could have their potential application as a more precise risk marker for STEMI patients undergoing pPCI.

### 4.1. Study limitations

The study population in this work was relatively small and the duration of the follow-up period was limited to one year after STEMI. Additionally, the number of the investigated events was relatively low. Furthermore, our data assessed the prognostic information of the presence of MVO and IMH but did not evaluate the extent of MVO and IMH. It would thus be important for future studies to elucidate the percentage of MVO in LV. CMR was performed on two days in STEMI patients who undergo pPCI. Yet, the IMH evaluation could have been affected due to its temporal variations in the few days after infarction.

Serum GSDMD levels were measured once, within 48 h post-reperfusion, the median time of GSDMD measurement was 28 h. Therefore, the GSDMD release dynamics over time (pre- and post-PCI) and its potential prognostic information could not be provided. However, our other data suggest that the inflammatory factor IL-1β reaches its peak at 48 h post-reperfusion and this might be the optimal time point of measurement. Nevertheless, the optimal timing of GSDMD measurements remains unknown in our study and needs further research. Moreover, the promising gasdermins family (GSDMC and GSDME) was not investigated in the present study. Thus, future investigations are required to evaluate the association between GSDMC or GSDME and MVO or IMH in STEMI patients receiving pPCI.

## 5. Conclusion

The increased serum levels of GSDMD measured at 48 h in STEMI patients treated with pPCI were independently associated with a higher MVO and IMH rates and larger myocardial injury as established by comprehensive CMR imaging. Moreover, the present results emphasize the value of GSDMD as a biomarker for risk stratification, which can serve as a potential therapeutic target for STEMI patients with microvascular post-reperfusion injury.

## Data Availability

The raw data supporting the conclusions of this article will be made available by the authors, without undue reservation.
