# Supplementary material for: Association of GSDMD with microvascular-ischemia reperfusion injury after ST-elevation myocardial infarction
Source: Front Cardiovasc Med. 2023 Jun 6;10:1138352. doi: 10.3389/fcvm.2023.1138352 (PMC10325858; doi:10.3389/fcvm.2023.1138352)
Supplement: Supplementary file 1 [file Datasheet1.docx]

**SUPPLEMENTAL MATERIAL**

**CMR protocol**

CMR was performed at 2 days and 12months after STEMI using a 3.0 T clinical scanner (Philips Ingenia 3.0 T CX [Philips Medical Systems Nederland B.V. Best, the Netherlands], the gradient field strength of 80 mT/m, gradient switching rate of 200 mT/m/ms equipped with a Q- BODY coil, SENSE XL TORSO COIL 3.0 T for signal reception). The CMR protocol has been previously described [17].

*Delayed hyperenhancement*

Delayed hyperenhancement was acquired 10-15min after intravenous injection of gadolinium diethylenetriamine penta-acetic acid (Gd-DTPA; 0.1mmol/kg) to identify infracted myocardium using (PSIR_TFE_BH) sequence; M2-Dmode; FFE, Single trigger. Scan parameters were as follows: THE factor: 19, TR/TE; 6.1/3.0ms; flip angle, 25°; FOV: 320 × 320mm; slice thickness: 8mm.

*Cine*

Short axis cine images covering the length of the ventricle were acquired as previously reported [19]. Cine-MR imaging was performed with balanced steady-state free precession (b-SSFP) along the horizontal long-axis, the vertical long-axis, the 3-chamber view, images covering the LV from the base to the apex repetition time/echo time 3.0 ms/1.5 ms; flip angle 60°; matrix 256 × 256; field of view 320 × 270 mm; slice thickness: 8mm.

*Native T1 Mapping*

Native mapping images were acquired with a steady-state free precession-based modified Look-Locker Inversion Recovery (MOLLI) sequence using a 5-3-3 modified MOLLI protocol. The acquisition parameters were flip angle, 35°; repetition time: 2.6ms; matrix: 256×144; echo time: 1.1 ms; slice thickness: 8 mm.

*T2* Mapping*

T2* mapping was performed before administration of the contrast agent by using a breath-hold, multiecho gradient echo (ME‐GRE) sequence with eight echoes obtained in three matching short-axis slices. Imaging parameters were: echo times×8: 2.7, 5, 7.3, 9.6, 11.9, 14.2, 16.5, and 18.8 ms; flip angle, 20°; matrix: 256×115; and slice thickness: 8 mm.

**Image Analysis**

All image analyses were carried out by two experienced radiologists using CV142 imaging software (Version 5.1.0 [280], Calgary, Canada). Measurements were performed by two experienced observers. End systolic volume (ESV) and end diastolic volume (EDV) and LVEF were calculated by manually tracing the endocardial outline of the short axis cine images. IS was quantified following manual delineation of the endocardium and epicardium of short axis slices and as a percentage of the LV (%LV) using 5 standard deviations (SD) threshold above the mean remote myocardium. The presence of MVO was defined as hypoenhancement within an hyperenhancement region in STEMI patients on the LGE images. Presence of IMH was identified in a computer-assisted motion corrected T2^*^ maps and defined as a hypointense core with a T2^*^ value of < 20ms. For T1 mapping, a ROI was manually drawn in hyper- intensity area using 2 SD threshold from the remote myocardium.

**Table S1. Cox Regression Analysis of the Primary Endpoint by CMR**


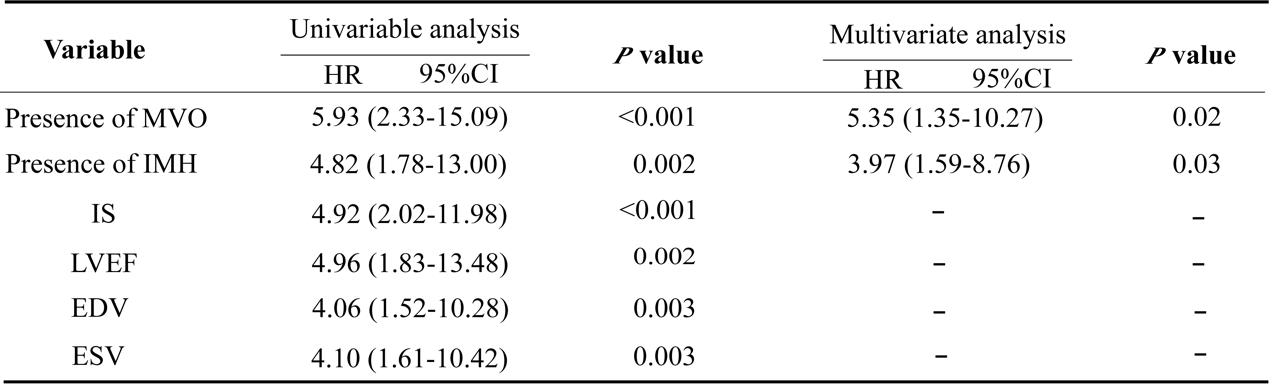


**MACE, major adverse cardiovascular event; LVEF, left ventricular ejection fraction; EDV, end-diastolic volume; ESV, end-systolic volume; IS, infarction size; MVO, microvascular obstruction; IMH, intramyocardial hemorrhage. The 2-tailed *P* value reaches statistical significance, *P*<0.05.**
